# Identification of Novel Interacting Partners of Sirtuin6

**DOI:** 10.1371/journal.pone.0051555

**Published:** 2012-12-11

**Authors:** Oxana Polyakova, Satty Borman, Rachel Grimley, Jessica Vamathevan, Brian Hayes, Roberto Solari

**Affiliations:** 1 Platform Technology Sciences, GlaxoSmithKline, Stevenage, Hertfordshire, United Kingdom; 2 Computational Biology, GlaxoSmithKline, Stevenage, Hertfordshire, United Kingdom; 3 Allergic Inflammation Discovery Performance Unit, GlaxoSmithKline, Stevenage, Hertfordshire, United Kingdom; Wake Forest University, United States of America

## Abstract

SIRT6 is a member of the Sirtuin family of histone deacetylases that has been implicated in inflammatory, aging and metabolic pathways. Some of its actions have been suggested to be via physical interaction with NFκB and HIF1α and transcriptional regulation through its histone deacetylase activity. Our previous studies have investigated the histone deacetylase activity of SIRT6 and explored its ability to regulate the transcriptional responses to an inflammatory stimulus such as TNFα. In order to develop a greater understanding of SIRT6 function we have sought to identify SIRT6 interacting proteins by both yeast-2-hybrid and co-immunoprecipitation studies. We report a number of interacting partners which strengthen previous findings that SIRT6 functions in base excision repair (BER), and novel interactors which suggest a role in nucleosome and chromatin remodeling, the cell cycle and NFκB biology.

## Introduction

Sirtuins are a family of proteins that appear to be involved in many cellular responses to stress, ranging from chromatin modification, genomic stability, metabolism, inflammation, cellular senescence and organismal lifespan and consequently have generated significant interest as potential therapeutic targets. Sirtuins are highly conserved through evolution and in mammals there are 7 members of the Sirtuin family (SIRT1-7) that can be grouped into four classes based on sequence alignments and SIRT6 and SIRT7 fall into the class IV group [1]. The seven mammalian Sirtuins have distinct cellular locations including the cytoplasm, mitochondria, nucleus and nucleolus. Sirtuins have been shown to catalyse two different NAD^+^ dependent reactions namely deacetylation and ADP-ribosylation. SIRT6 has been shown to possess both activities but to date most of the biological functions of SIRT6 have been ascribed to its deacetylase activity against a small set of substrates which include acetylated Histone 3 Lysine 9 (H3K9Ac) [2], acetylated Histone 3 Lysine 56 (H3K56Ac) [3], [4] and CtIP [5], although SIRT6 has additionally been shown to ADP-ribosylate itself [6] and PARP1 [7]. SIRT6 is mainly localized to the nuclear matrix associated with histones, based on immunocytochemistry [6], [8] and subcellular fractionation studies [8] and excluded from the nucleolus whereas SIRT7 is a nucleolar protein [9]. More recent detailed analysis has shown SIRT6 can also be detected in the nucleolus, particularly at the G1 phase of the cell cycle [10].

The clearest biological function of SIRT6 so far appears to be in the maintenance of genome integrity which has largely been deduced from the phenotype of knockout mice and cells where SIRT6 levels have been knocked down with siRNA or shRNA [2], [8]. SIRT6 knockout mice appear normal at birth but have a greatly shortened lifespan and show degenerative and metabolic defects reminiscent of premature aging syndromes [8]. In addition, SIRT6 deleted embryonic stem cells and mouse embryonic fibroblasts have impaired proliferation and increased sensitivity to DNA-damaging agents and showed a number of chromosomal abnormalities [8]. Double strand break (DSB) repair and cell cycle checkpoint appeared normal in these cells and it was shown that the sensitivity to DNA damage and enhanced genomic instability in SIRT6 knockout cells was consistent with a role in base excision repair (BER). More recent studies have gone on to show that SIRT6 is also involved in DSB repair by binding DNA-dependent protein kinase [11] and promoting DNA end resection through CtIP deacetylation [5] as well as by ADP-ribosylating and activating PARP1 [7]. With regards to genome stability it has also been shown by knocking down SIRT6 in cell lines with shRNA that SIRT6 is physically associated with telomeres and SIRT6 plays an important role in telomere function [2]. Depletion of SIRT6 led to premature cellular senescence, abnormal telomere structures and end-to-end chromosomal fusions suggesting loss of normal telomere function.

SIRT6 has recently been discovered to have an additional function as a transcriptional regulator through post-translational modification and physical interaction with the transcription factors NFκB [12] and HIF1α [13]. This is not unique to SIRT6 and SIRT1, another nuclear Sirtuin, can also regulate gene expression by physical interaction and deacetylation of HIF1α at Lys674 which blocked p300 recruitment and so suppressed HIF1α target genes [14]. SIRT1 is also known to suppress NFκB function through binding to RelA/p65 and deacetylation of Lys310 [15] and SIRT2, which is a cytoplasmic Sirtuin, also physically interacts with NFκB and suppresses its actions through deacetylation of Lys310 [16]. Both SIRT6 and SIRT7 have been shown to physically interact with the NFκB RelA/p65 subunit [12] and activation of RelA/p65 was shown to recruit SIRT6 to chromatin of NFκB target genes where it deacetylates H3K9Ac, terminating NFκB signalling presumably through condensation of chromatin. Consequently it was proposed that SIRT6 may be a master regulator of glucose homeostasis in addition to a regulator of inflammatory gene expression and thus provide an attractive mechanistic explanation for the link between inflammation and aging. This connection between SIRT6 and the function of NFκB is potentially of great therapeutic importance, however we were unable to show that over expression of wild type or a catalytically dead mutant of SIRT6 had any significant influence on the profile of NFκB dependent gene expression induced by stimulation of cells with TNFα [17]. Since our present study was aimed at identifying SIRT6 interacting proteins, we first wished to confirm the reported interaction between RelA/p65 and SIRT6.

In our previous studies we characterised the catalytic activity of purified SIRT6 by *in vitro* biochemical assays [17]. Although the histone deacetylase activity of SIRT6 has clearly been demonstrated in cells and tissues in numerous studies [2]–[5], [7], [13], [14], [18], its *in vitro* catalytic activity, albeit in non-quantitative assays, had been shown as either modest [2], [3] or absent [6]. Careful quantitative enzymological analysis by our group and others has shown that purified SIRT6 has an extremely low catalytic activity *in vitro* [17], [19]. We found a *k_cat_* of 1.67×10^−5^ s^−1^ and a *K_m, app_* for the peptide substrate of 14 µM thus giving a *k_cat_/K_m_* ratio of 1.2 M^−1^ s^−1^ which is five orders of magnitude lower than the median *k_cat_/K_m_* ratio of 1.25×10^5^ **M^−1^ s^−1^ determined by examining nearly 2000 enzymes [20]. Nevertheless, we were able to confirm, as others had previously shown, that SIRT6 demonstrated H3K9Ac deacetylase activity when transfected and overexpressed in cells [17]. This led us to suspect that SIRT6 required other factors, components or modifications in order for it to display its full cellular deacetylase activity. Consequently to address these questions we sought to identify SIRT6 binding proteins by yeast-2-hybrid and proteomics techniques. We report here the initial findings in our search for SIRT6 interacting partners.

## Results

### SIRT6 Interaction with RelA/p65

**Figure 1:**
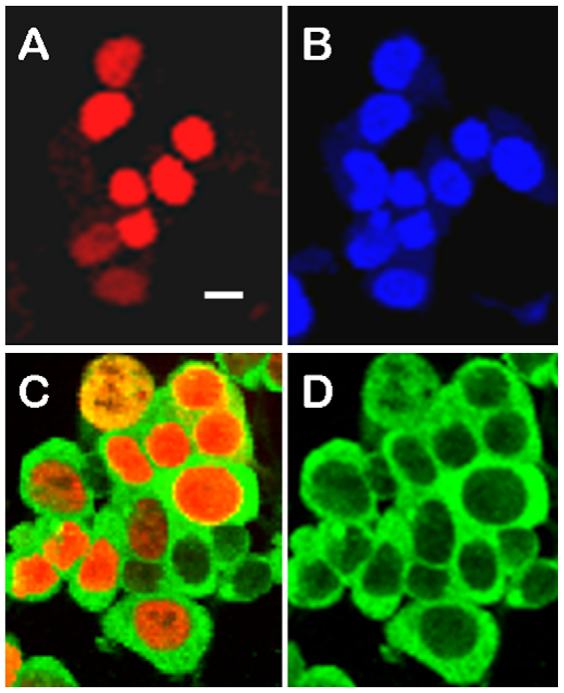
HEK293 cells were transiently transfected with Flag-tagged SIRT6 and analysed by immunocytochemistry and confocal microscopy. Panel A shows anti-Flag staining (red) and panel B the same cells counterstained with Hoechst33342 (blue). Panel C shows cells double stained with anti-RelA/p65 (green) and anti-Flag (red) and panel D only shows the corresponding anti-RelA/p65 staining alone. Transfected and untransfected cells showed similar gross morphology and clear nuclear localization of Flag-SIRT6. Scale bar = 10 µm.

**Figure 2:**
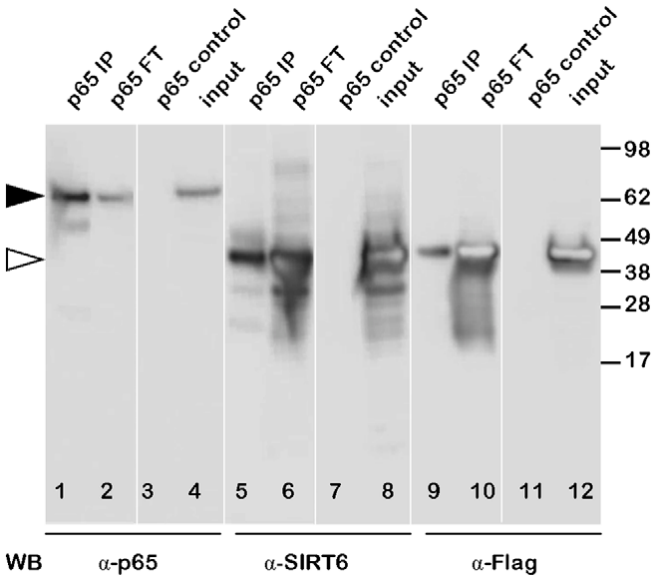
Interaction of SIRT6 with RelA/p65. HEK293 cells were transiently transfected with Flag-tagged SIRT6. Cell extracts were prepared and immunoprecipitated with an antibody to RelA/p65 and analysed by Western blotting (WB) with antibodies to RelA/p65 (lanes 1–4) SIRT6 (lane 5–8) and Flag (lane 9–12). Lanes 1,5 and 9 show the anti-RelA/p65 immunoprecipitate (p65 IP), lanes 2,6 and 10 show the non-immunoprecipitated or “flow through” material (FT), lanes 3,7 and 11 show a control immunoprecipitation using only Protein G agarose (p65 control) and lanes 4,8 and 12 are a sample of the total cell extract (input).

**Figure 3:**
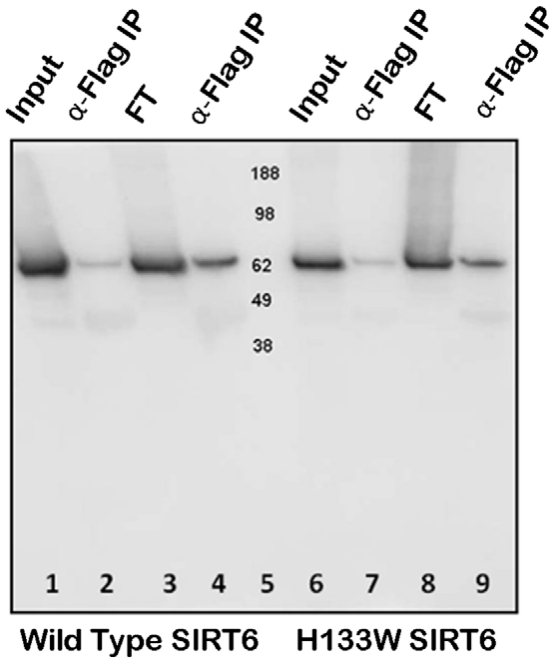
HEK293 cells were transiently transfected with Flag-tagged wild type SIRT6 (lanes 1–4) or the Flag-tagged H133W mutant (lanes 6–9). Cell extracts were prepared and immunoprecipitated with an anti-Flag antibody and analysed by Western blotting with anti-RelA/p65 antibody. Lanes 1 and 6 represent the total cell extracts used in the immunoprecipitation (input). Lanes 2 and 7 represent the anti-Flag immunoprecipitation eluted from the beads with Flag peptide (α-Flag IP). Lanes 3 and 8 represent the non-immunoprecipitated “flow through” material from the cell extract (FT) and lanes 4 and 9 represent the immunoprecipitated material eluted from the beads with SDS-PAGE sample buffer (α-Flag IP). Lane 5 contains molecular mass markers.

SIRT6 has been proposed to be a repressor of both NFκB and HIFα regulated gene expression via a physical interaction and recruitment to promoters and subsequent deacetylation of chromatin. We therefore sought to study SIRT6 binding partners by two independent protein interaction mapping techniques. As our methods rely on transient transfection studies where we overexpress SIRT6, we first controlled that the transfected SIRT6 showed clear nuclear localisation and did not induce gross morphological changes to cells. Figure 1 shows HEK293 cells transiently transfected with Flag-tagged SIRT6 and double labelled with anti-Flag with either Hoechst33342 stain or with anti-RelA/p65. In our transfection conditions it was clear that the overexpressed SIRT6 was nuclear and there was no obvious gross morphological change to the cells. Of interest to subsequent experiments Flag-SIRT6 and RelA/p65 under these unstimulated conditions do not show greatly overlapping localisation in the cell although it is well established that under unstimulated conditions a proportion of RelA/p65 is always nuclear. To confirm the previously described SIRT6 interaction with RelA/p65, Flag tagged SIRT6 was transiently transfected into HEK293 cells and cell extracts were subsequently immunoprecipitated with anti-RelA/p65 followed by Western blotting and immunodetection with anti-RelA/p65, anti-SIRT6 and anti-Flag (Figure 2). Lane 1 shows the RelA/p65 immunoprecipitate followed by anti-RelA/p65 Western blotting. Lane 2 shows the non-immunoprecipitated material (flow through or FT) and lane 3 a control immunoprecipitation using only Protein-G agarose and omitting the anti-RelA/p65 antibody and finally lane 4 shows a Western blot of a whole cell extract (RelA/p65 shown by black arrowhead). The same samples were also Western blotted with anti-SIRT6 (lanes 5–8) and with anti-Flag (lanes 9–12, SIRT6 shown by open arrow head). It was clear from this experiment that immunoprecipitation of RelA/p65 from cell extracts clearly pulled down RelA/p65 and co-immunoprecipitated SIRT6 as detected with either anti-Flag or anti-SIRT6. We confirmed and extended this observation by transfecting HEK293 cells with either wild type or the catalytically dead H133W mutant of SIRT6 and reversing the protocol by immunoprecipitating the cell extract with anti-Flag followed by Western blotting with anti-RelA/p65 (Figure 3). For comparisons we loaded the total cell extract in lanes 1 and 6. Cell extracts were immunoprceipitated with anti-Flag antibody and bound proteins eluted from the beads with Flag peptide. This mild bead elution released small but detectable amounts of RelA/p65 (lanes 2 and 7). Lanes 3 and 8 show the non-immunoprecipitated (flow through - FT) material from the cell extract and lanes 4 and 9 represent the immunoprecipitated material eluted from the beads with SDS-PAGE sample buffer. From these results it is clear that both the wild type and H133W are equally effective at co-immunoprecipitating RelA/p65 and that a substantial proportion of the total cellular pool of RelA/p65 can be pulled down with SIRT6.

### SIRT6 Yeast-two-hybrid Screen

**Figure 4:**
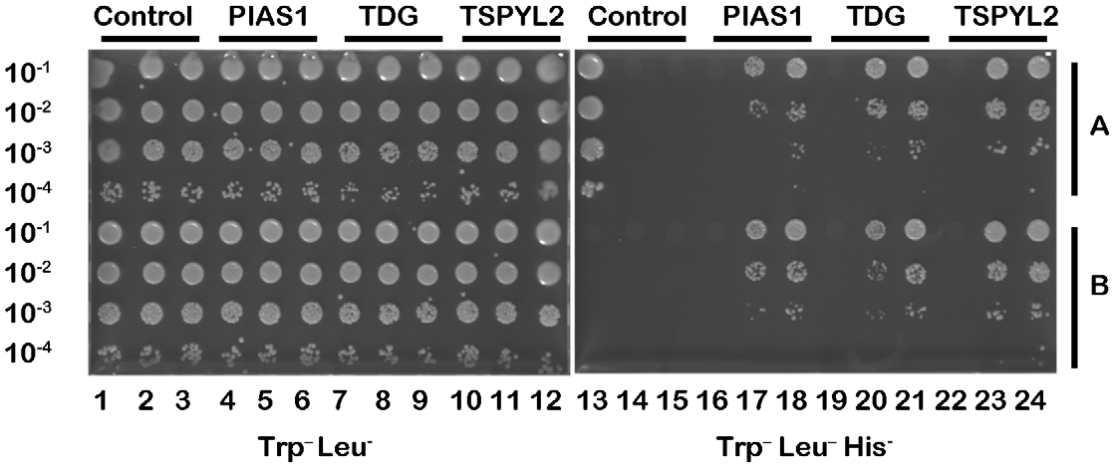
Yeast-two-hybrid analysis. The coding sequence for full length wild type SIRT6 and the H133W mutant was PCR-amplified and cloned in frame with the LexA DNA binding domain (DBD) into the bait plasmid pB27. Fragments corresponding to amino acids 303–507 of PIAS1, amino acids 101–410 of TDG and amino acids 166–287 of TSPYL2 were extracted from the ULTImate Y2H™ human leukocyte and activated mononuclear cell library and cloned into the pB6 prey plasmid. Interactions were tested in growth assays as two independent clones (A and B) picked from each co-transformation except for the HGX positive control (columns 1 and 13, block A) and the empty controls (empty bait pB27 vector and empty prey vector pB6 columns 1 and 13, block B) that were only tested as a single clone. For each interaction several dilutions (10^−1^, 10^−2^, 10^−3^ and 10^−4^) of the diploid yeast culture normalized at 5×10^4^ cells and expressing both bait and prey constructs were spotted on the selective media. The left hand plate shows growth on media lacking tryptophan and leucine as a growth control test and to verify co-transformation of both plasmids. The same dilutions were spotted onto medium lacking tryptophan, leucine and histidine to confirm interaction of the bait and prey (right hand plate). For each interaction there is an empty bait (C-pB27) control co-transformation (columns 4, 7 and 10 for left hand plate and columns 16, 19 and 22 for the right hand plate). Column 5, 8, 11, 17, 20 and 23 have the H133W mutants cloned into the C-pB27 bait plasmid and columns 6, 9, 12, 18, 21 and 24 have the wild type SIRT6 cloned into the C-pB27 bait plasmid.

Having confirmed that SIRT6 and RelA/p65 can be shown to physically interact by co-immunoprecipitation, we extended our analysis of interacting proteins by performing a yeast-two-hybrid study using a mating assay protocol as described in Materials and Methods. Two constructs were made with full length SIRT6 as bait (residues 1–355) fused C-terminal to either LexA or Gal4 DNA binding domains and both baits were tested against a human leukocyte/activated mononuclear cell library as described. Extensive library screening with both baits identified the same 3 preys; protein inhibitor of activated STAT1 (PIAS1), thymine DNA glycosylase (TDG), and a splice variant transcript of TSPYL2. The interacting fragments of the three preys were residues 303–507 for PIAS1 (gi: 7706636), residues 101–410 for TDG (gi:197927092) and residues 166–287 for TSPYL2 (gi:47076936). These three cDNAs were re-sequenced and cloned back into the prey plasmid and the SIRT6 interactions confirmed in one-by-one assays (Figure 4). All three interacting proteins confirmed binding to the H133W and wild type SIRT6 baits and the binding was equally sensitive to 3-Aminotriazole (data not shown). The alternative splice form of TSPYL2 has a predicted ORF of 287 amino acids (aa), the first 269 aa of which are identical to the first 269 aa of the major splice form of TSPYL2 (gi:259906401). The C-terminus of the 287 aa ORF encodes a partial NAP domain and from previous yeast-2-hybrid screens it is known that this NAP domain corresponding to residues 166–287 of TSPYL2 also interacts with p53 (Hybrigenics personal communication). Hybrigenics have to date performed greater than 4500 screens with distinct human baits against 25 different libraries and the only two interactors with TSPYL2 are SIRT6 and p53. PIAS1 is a SUMO ligase and well known transcriptional regulator of STAT1 and NFκB and is known to interact with many proteins including p53. The clone identified as a SIRT6 binder (residues 303–507) includes the Zn finger domain of PIAS1 (320–397), the nuclear localization sequence (368–380) and the SUMO binding domain (462–473). PIAS1 was found in 111 different screens performed at Hybrigenics on any of their human libraries, including 7 different baits against the human leukocyte library, two of which were SIRT6 and p53. TDG was found in 34 different screens performed at Hybrigenics across all their libraries but in the human leukocyte library the only TDG interactors are SIRT6 and p53 both of which bound the same aa 101–410 region of TDG. RelA/p65 did not appear as a SIRT6 interactor in this yeast two hybrid assay.

### SIRT6 Proteomics

In a parallel approach we overexpressed Flag-tagged SIRT6 in HEK293 cells and immunoprecipitated SIRT6 and any bound proteins with an anti-Flag antibody. The protein complex was run on a SDS PAGE and Coomassie stained. A number of co-immunoprecipitated bands were visualized, excised and sequenced by mass spectrometry and we report on two of those proteins in this study. A band with an apparent molecular mass of 150 kDa was analysed by LC MS/MS and generated six peptides SPLSALAR, LITGLGVGR, SPSLLQSGAK, VVVTDDSDER, EIPSATQSPISK and KSEDGTPAEDGTPAATGGSQPPSMGR which were searched in a non-redundant protein sequence database using MASCOT (Table 1). This search revealed all six peptides correspond to the sequence of the MYB-binding protein 1A (MYBBP1A) (gi:157694492). MYBBP1A has been reported to be a repressor of NFκB and to directly bind to it [21]. A second band with an apparent molecular mass of 123 kDa was analysed by LC MS/MS in the same way generated a further four peptides, EIQEPDPTYEEK, RTEQEEDEELLTESSK, LVDQNLNK and ESEITDEDIDGILER (Table 1). Searching of the protein sequence database using MASCOT revealed all four peptides correspond to the sequence of the SMARCA5 protein (gi:325651836) a component of the SWI/SNF complex. Alignment of the peptides with the full length protein sequences is shown in Figure S1.

**Table 1:** Proteins co-immunoprecipitated with SIRT6 were analysed by MALDI-TOF Mass Spectrometry.

Ion (*m/z*)	Sequence	Description	Swiss-Prot Accession	Mascot E value
472.3739.3867.4641.6	LVDQNLNKEIQEPDPTYEEKESEITDEDIDGILERRTEQEEDEELLTESSK	SMARCA 5	O60264	0.361.25.7e-067.3e-04
407.7443.2494.3567.7629.3839.6	SPLSALARLITGLGVGRSPSLLQSGAKVVVTDDSDEREIPSATQSPISKKSEDGTPAEDGTPAATGGSQPPSMGR	Myb-binding protein 1A	Q9BQG0	5.7e-032.63.6e-021.1e-032.29.5e-03

Mass spectra were collected and individual peptide ions were selected for fragmentation analysis by LIFT-MS/MS sequencing. Spectra were interpreted as described and the data searched against protein sequence databases using the Mascot programme. The table shows the detected ion, the corresponding peptide, the name and accession number of the protein in which that sequence is found and the Mascot E value reflecting confidence of the assignment. M = Methionine oxidised.

### Co-immunoprecipitation of SIRT6 Binding Partners

**Figure 5:**
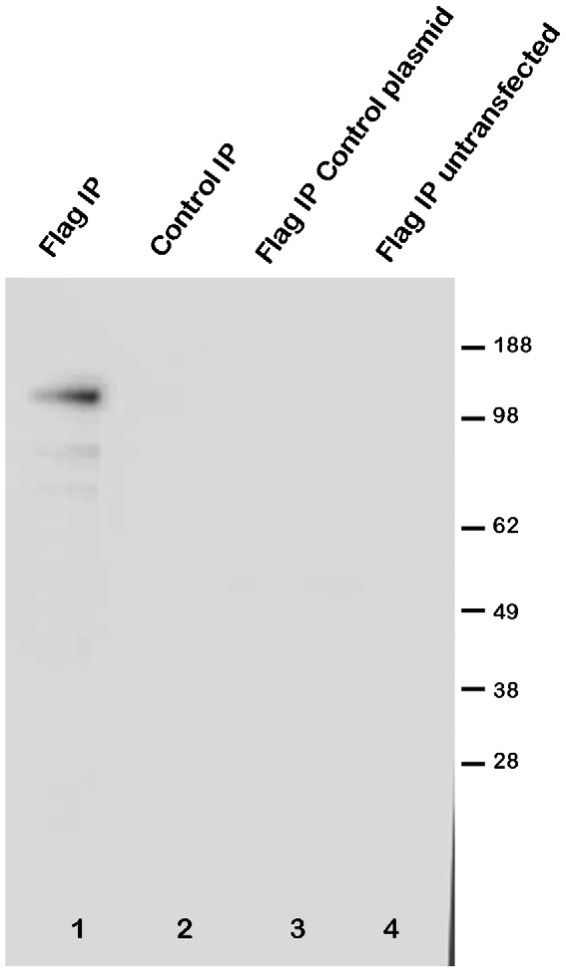
Confirmation of the interaction of SIRT6 with MYBBP1A. HEK293 cells were transiently transfected with Flag-tagged SIRT6. Cell extracts were prepared and immunoprecipitated with an antibody to Flag followed by Western blotting with an antibody to MYBBP1A (lane 1 Flag IP). As a “no antibody” control the cell extract was immunoprecipitated with no anti-Flag antibody but with Protein G agarose alone (lane 2 control IP). As additional controls, cell extracts were also immunoprecipitated with anti-Flag antibody from cell extracts prepared from cells transfected with a control empty plasmid (lane 3 Flag IP control plasmid) or extracts were immunoprecipitated with anti-Flag from cell extracts prepared from untransfected cells (lane 4 Flag IP untransfected).

**Figure 6:**
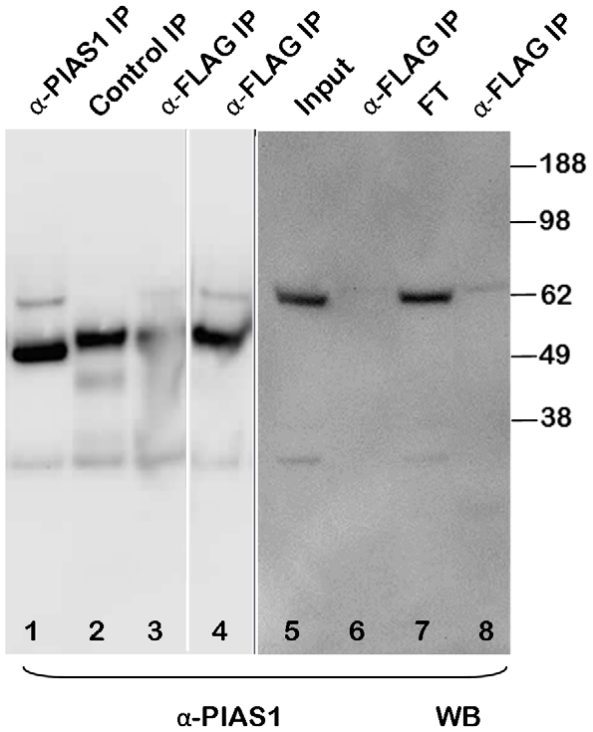
PIAS1 SIRT6 co-immunoprecipitation. HEK293 cell extracts were prepared and immunoprecipitated with an antibody to PIAS1 (lane 1 αPIAS1 IP) or an irrelevant antibody (lane 2 Control IP) followed by Western blotting with anti-PIAS1 antibody. The PIAS1 band was seen to migrate just above the 62 kDa marker. HEK293 cells were transiently transfected with Flag-tagged wild type SIRT6 (lane 3) or the H133W mutant (lane 4) and immunoprecipitated with anti-Flag followed by Western blotting with anti-PIAS1 (α-Flag IP). In a separate experiment HEK293 cells were transiently transfected with Flag-tagged wild type SIRT6 and cell extracts were prepared and immunoprecipitated with an anti-Flag antibody and analysed by Western blotting with anti-PIAS1 antibody (lanes 5–8). Lane 5 represents the total cell extract used in the immunoprecipitation (input). Lane 6 represents the anti-Flag immunoprecipitation eluted from the beads with Flag peptide (α-Flag IP). Lanes 7 represents the non-immunoprecipitated “flow through” material from the cell extract (FT) and lanes 8 represents the immunoprecipitated material eluted from the beads with SDS-PAGE sample buffer (α-Flag IP).

**Figure 7:**
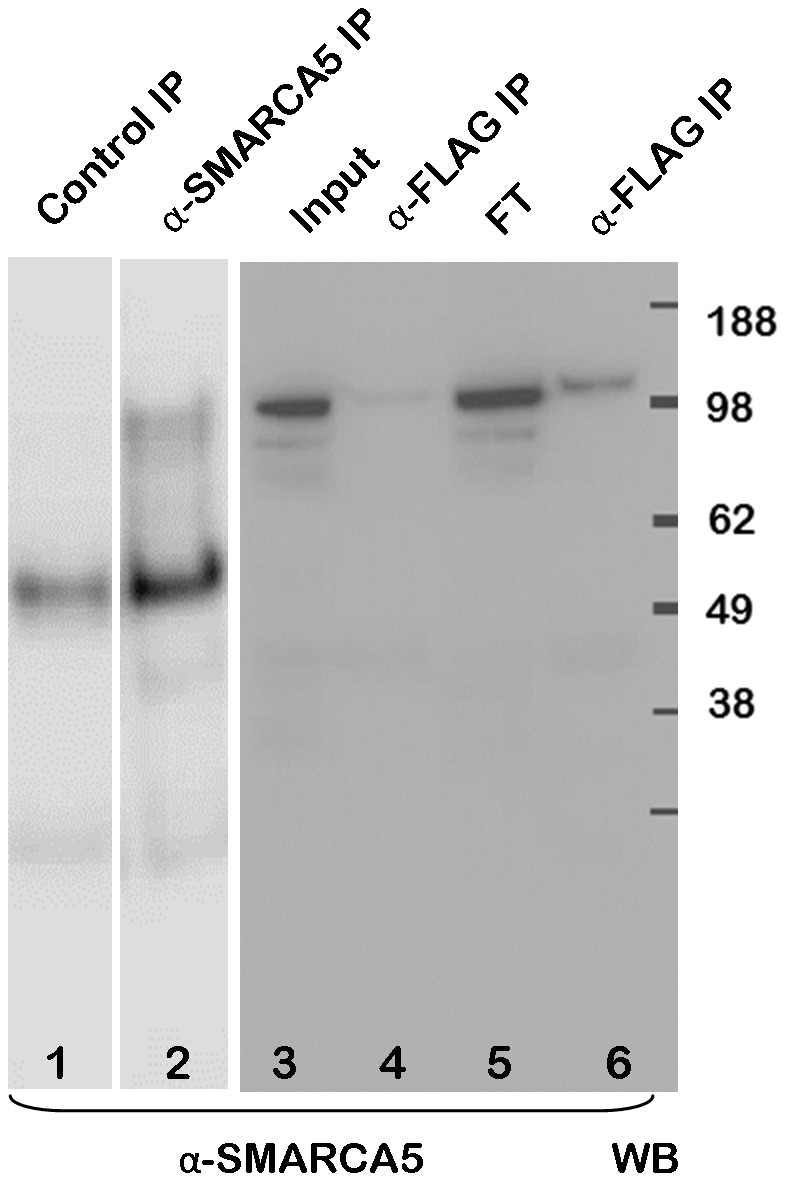
SMARCA5 SIRT6 co-immunoprecipitation. HEK293 cells were transiently transfected with Flag-tagged wild type SIRT6. Cell extracts were prepared and immunoprecipitated with an irrelevant antibody (lane 1 Control IP) or an antibody to SMARCA5 (lane 2 αSMARCA5 IP). The SMARCA5 band was seen to migrate above the 98 kDa marker. In a separate experiment HEK293 cells were transiently transfected with Flag-tagged wild type SIRT6 and cell extracts were prepared and immunoprecipitated with an anti-Flag antibody and analysed by Western blotting with anti-SMARCA5 antibody (lanes 3–6). Lane 3 represents the total cell extract used in the immunoprecipitation (input). Lane 4 represents the anti-Flag immunoprecipitation eluted from the beads with Flag peptide (α-Flag IP). Lanes 5 represents the non-immunoprecipitated “flow through” material from the cell extract (FT) and lanes 6 represents the immunoprecipitated material eluted from the beads with SDS-PAGE sample buffer (α-Flag IP).

To confirm these novel protein interactions of SIRT6 we performed further co-immunoprecipitation experiments. In the first study to confirm the MYBBP1A interaction, HEK293 cells were transfected with Flag-tagged SIRT6 and cell extracts were subsequently immunoprecipitated with an anti-Flag antibody (Figure 5 lane 1) or control immunoprecipitated using Protein G agarose beads without anti-Flag antibody (lane 2). As additional controls, extracts were made from cells that were transfected with an empty plasmid (lane 3) or were untransfected (lane 4). The immunoprecipitates were analyzed by Western blotting and detection with antibodies to MYBBP1A. This experiment appears to confirm that MYBBP1A can be specifically immunoprecipitated with SIRT6 from Flag-SIRT6 transfected cells. We went on to confirm additional interactions. To control for PIAS1, HEK293 cell extracts were immunoprecipitated with anti-PIAS1 followed by Western blotting with anti-PIAS1 (Figure 6 lane 1). A control immunoprecipitate was performed using an irrelevant antibody (Figure 6 lane 2). This clearly revealed a specific band at approximately 70 kDa, close to the expected mass of PIAS1. Extracts were also made from HEK293 cells overexpressing either wild type or the H133W SIRT6 mutant and immunoprecipitated with anti-Flag followed by Western blotting with anti-PIAS1 (lanes 3 and 4). In both cases the anti-Flag antibody was able to co-immunoprecipitate PIAS1. In a repeat experiment wild type Flag-SIRT6 plasmid was transfected into HEK293 cells and a total cell extract was loaded in lane 5. An equivalent amount of cell extract was also immunoprecipitated with anti-Flag and gently eluted from the beads with Flag peptide (lane 6) and the non-immunoprecipitated flow through was loaded in lane 7. An equivalent cell extract was anti-Flag immunoprecipitated but eluted from the beads with SDS-PAGE sample buffer (lane 8) and all samples were Western blotted with anti-PIAS1. This study confirms that PIAS1 can be pulled down effectively with anti-PIAS1 and anti-SIRT6. Similarly we controlled for SMARCA5 by immunoprecipitating an HEK293 extract using an irrelevant control antibody (Figure 7 lane 1) or with anti-SMARCA5 antibody (lane 2) followed by Western blotting with anti-SMARCA5 antibody. This revealed a band migrating with the expected apparent molecular mass slightly above 100 kDa. Wild type Flag-SIRT6 plasmid was transfected into HEK293 cells and a total cell extract was loaded in lane 3. The cell extract was also immunoprecipitated with anti-Flag and eluted from the beads with Flag peptide (lane 4) and the non-immunoprecipitated flow through was loaded in lane 5. An equivalent cell extract was anti-Flag immunoprecipitated but eluted from the beads with SDS-PAGE sample buffer (lane 6) and all immunoprecipitates were Western blotted with anti-SMARCA5 antibody. These results clearly show that SIRT6 can co-immunoprecipitate SMARCA5 under these conditions.

**Figure 8:**
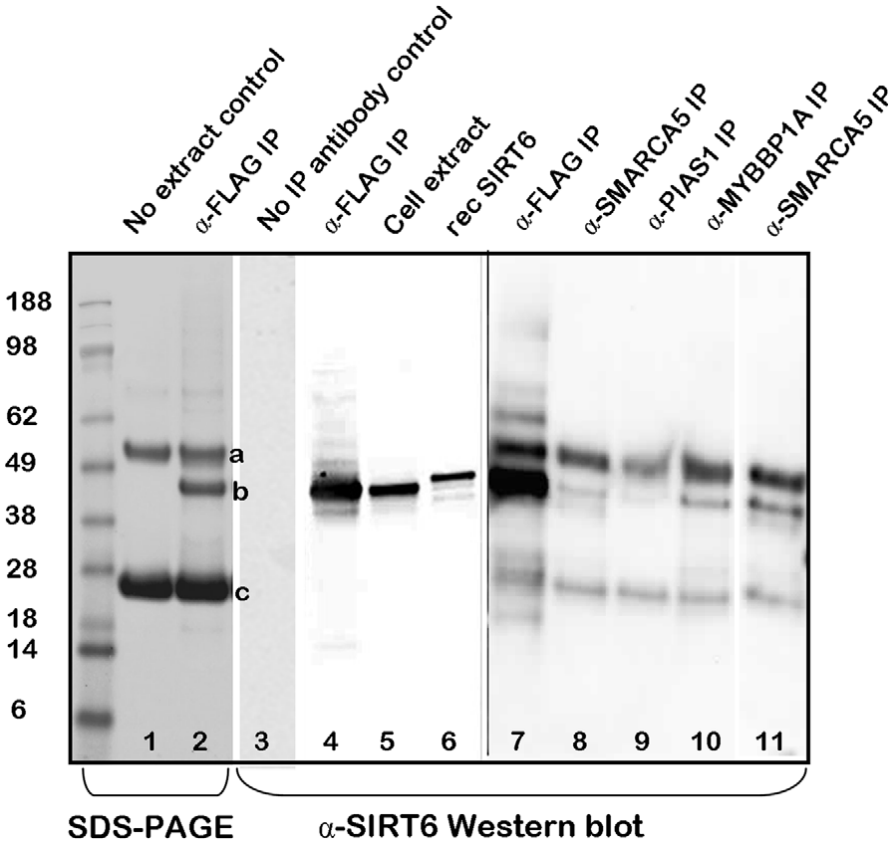
HEK293 cells were transiently transfected with Flag-tagged wild type SIRT6 and cell extracts were prepared and immunoprecipitated with a variety of antibodies and analysed by Coomassie staining the SDS-PAGE (lanes 1,2) or by Western blotting with anti-SIRT6 (lanes 3–11). Lanes 2 shows a stained SDS-PAGE of a HEK293 cell extract immunoprecipitated with anti-Flag (α-Flag IP) and lane 1 shows a control where the extract was omitted (no extract control). The three bands seen in lane 2 (a,b,c) were excised and sequenced by ms (Table 2). Lanes 3–11 are all anti-SIRT6 Western blots. Lane 3 shows a control immunoprecipitation where the anti-Flag antibody was omitted from the IP (no IP antibody control). Lane 4 shows the anti-Flag immunoprecipitate and lane 5 shows a sample of the total cell extract without immunoprecipitation. Lane 6 shows a sample (150 ng) of purified recombinant SIRT6 [17]. In a separate experiment and at higher exposure of the Western blot, lane 7 shows an anti-Flag IP, lane 8 shows an anti-SMARCA5 IP, lane 9 shows an anti-PIAS1 IP, lane 10 shows an anti-MYBBP1A IP and lane 11 shows an anti-SMARCA5 IP from H133W transfected cells. The figure is a composite of more than one experiment but represents data from three repeated experiments.

We went on to reverse the study by transfecting HEK293 cells with Flag-tagged SIRT6 and immunoprecipitating the cell extracts with antibodies to endogenous SMARCA5, PIAS1, MYBBP1A and SIRT6 and Western blotting with anti-SIRT6 (Figure 8). Whereas detecting SIRT6 by Western blotting an anti-Flag immunoprecipitate was straightforward, detecting SIRT6 as a co-immunoprecipitate with other antibodies was more challenging and required higher exposures of the blot to reveal the specific SIRT6 band. These higher exposures often revealed additional bands on the blot and therefore we sought to carefully control this experiment. Firstly we immunoprecipitated with anti-Flag a large scale extract from HEK293 cells that had been transfected with Flag-SIRT6, and ran the immunoprecipitate on an SDS-PAGE followed by Coomassie staining. In lane 1 there is a control immunoprecipitate with anti-Flag and Protein G agarose beads but no cell extract and in lane 2 there is the anti-Flag immunoprecipitated extract. The bands (a,b,c) at 55 kDa, 40 kDa and 25 kDa were excised and sequenced and their identity was revealed to be mouse immunoglobulin heavy chain, SIRT6 and mouse immunoglobulin light chain respectively (Table 2). In a separate experiment, we controlled for the anti-Flag antibody by omitting it from the immunoprecipitation protocol. The HEK293 cell extract was incubated with control agarose beads without anti-Flag antibody, followed by extraction and analysis of the sample by Western blotting with anti-SIRT6 (lane 3). We also immunoprecipitated the HEK293 extract with anti-Flag and Western blotted with anti-SIRT6 (lane 4) and also ran as controls a sample of the whole cell extract (lane 5) and a sample of recombinant Flag-His-tagged SIRT6 protein (lane 6). In a separate experiment we immunoprecipitated the HEK293 cell extract with anti-Flag (lane 7) anti-SMARCA5 (lane 8) anti-PIAS1 (lane 9) and anti-MYBBP1A (lane 10) followed by Western blotting with anti-SIRT6. We also transfected HEK293 cells with the H133W mutant and immunoprecipitated the cell extract with anti-SMARCA5 (lane 11). Higher exposure of this blot revealed Immunoglobulin heavy and light chains, but also clearly revealed co-immunoprecipitation of SIRT6.

**Table 2:** HEK293 cell were transfected with Flag-tagged SIRT6 and extracts were immunoprecipitated with anti-Flag.

Band #	Ion(*m/z*)	Sequence	Description	Mascot E value
a	1243.671813.931981.86	VNSAAFPAPIEKAPQVYTIPPPKEQMAKNTQPIMDTDGSYFVYSK	ImmunoglobulinHeavy chains	0.0002130.00018
b	1283.811298.611460.791469.841483.851532.931574.771625.961800.881990.97	ALPPLPRPPTPKGPHGVWTMEERHLGLEIPAWDGPRERPTSPAPHRPPKFLVSQNVDGLHVRLVIVNLQPTKHDRDTILDWEDSLPDRRERPTSPAPHRPPKCGLPEIFDPPEELERDKLAELHGNMFVEECAK	Flag-6His-Th-TEV-SIRT6	0.0140.00071.9e-080.915.8e-060.00831.5e-060.000253.9e-090.022
c	2061.952600.32	FSGVPDRFSGSGSGTDFTLKDVLMTQIPLSLPVSLGDQASISCR	Immunoglobulin light chains	0.0425.2e-13

The proteins extracted from the anti-Flag/Protein G agarose beads were run on an SDS-PAGE and the gel Coomassie stained (Figure 8 lane 2). The three major bands were excised and analysed by MALDI-TOF Mass Spectrometry. Mass spectra were collected and individual peptide ions were selected for fragmentation analysis by LIFT-MS/MS sequencing. Spectra were interpreted as described and the data searched against protein sequence databases using the Mascot programme. The table shows the detected ion, the corresponding peptide and the name of the protein in which that sequence is found and the Mascot E value reflecting confidence of the assignment. M = Methionine oxidised.

## Discussion

The first biological functions ascribed to SIRT6 involved the maintenance of telomere integrity where it was shown to localize and deacetylate H3K9Ac. It was shown to be required for the stable association of the Werner’s syndrome protein with telomeric chromatin and loss of function of SIRT6 leads to damage to telomeres and premature cellular senescence [2]. These observations were the first link between SIRT6 and aging and cancer. SIRT6 knockout mice display a greatly shortened lifespan and acute degenerative and metabolic defects similar to premature aging pathologies. It was further shown that SIRT6 knockout embryonic stem cells and mouse embryonic fibroblasts showed impaired proliferation and increased sensitivity to DNA-damaging agents [8]. These studies demonstrated that SIRT6 promoted resistance to DNA damage and suppressed genomic instability consistent with a role in base excision repair (BER) although double strand break (DSB) repair and cell cycle checkpoint appeared normal. SIRT6 deficient cells were hypersensitive to DNA damage and this could be rescued by over expression of the dRP lyase domain of DNA polymerase β [8]. However failure to detect functional interactions between SIRT6 and components of the BER mechanism cast doubt over this conclusion [22]. Recent studies have gone on to show that SIRT6 is involved in DSB repair by forming a macromolecular complex with DNA-dependent protein kinase [11] and SIRT6 promotes DNA end resection through CtIP acetylation [5]. More recently SIRT6 has been shown to promote DNA repair by ADP-ribosylating and activating of PARP1 [7]. Subsequent studies revealed a new role for SIRT6 as a transcriptional regulator through direct physical interaction with NFκB and HIF1α and recruitment of this histone deacetylase to repress active chromatin sites. Our previous studies showed that the H3K9Ac deacetylase activity of purified SIRT6 *in vitro* was so low that we questioned whether this represented a real catalytic activity. However, when transfected into cells, SIRT6 did display an ability to deacetylate H3K9Ac suggesting that other cellular factors may be required for SIRT6 to be catalytically active. Moreover, we were unable to confirm in our studies that overexpression of either wild type or catalytically dead SIRT6 influenced NFκB dependent gene expression at 4 hours after TNFα stimulation [17].

There is a substantial body of evidence demonstrating the role of NFκB both in the activation of inflammatory gene expression and aging. Analysis of whole genome microarray studies found there was a strong pattern of expression with genes showing increased expression with age which consistently had an NFκB motif in their promoter [23]. Many stressors that reduce longevity activate NFκB and proteins known to be involved in longevity, SIRT1 and FOXO have been shown to inhibit NFκB. There is also direct experimental evidence linking NFκB to skin aging [24]. Further links between Sirtuins and NFκB are becoming clear. Resveratrol (described as a prototypical Sirtuin1 activator) has been reported to inhibit NFκB dependent transcription [25]–[27]. More recently, SIRT1 has been shown to deacetylate RelA/p65 protein at lysine 310 which inhibits NFκB activity and sensitises cells to TNFα induced apoptosis [15]. There is growing complexity in the post-translational modifications that have been shown to regulate NFκB, including phosphorylation, acetylation and methylation [28] and so there is considerable interest to understanding the contribution of various Sirtuins to NFκB function. SIRT2, which is a cytoplasmic Sirtuin, also physically interacts with NFκB and suppresses its actions through deacetylation of Lys310 [16] and both SIRT6 and SIRT7 have been shown to physically interact with the NFκB RelA/p65 subunit [12]. The suggestion that SIRT6 physically interacted with RelA/p65 and repressed its function led to an appealing mechanistic model that linked aging and inflammation. Although our previous studies failed to show an effect of over expression of SIRT6 or a catalytically dead mutant of SIRT6 on NFκB transcriptional responses to a TNFα stimulus, in our current study we confirm that SIRT6 and RelA/p65 clearly can be shown to co-immunoprecipitate from cell extracts. This suggests that if SIRT6 does indeed regulate the function of NFκB the biological mechanisms in which such regulation operates may be subtle and context specific.

Our previous findings that SIRT6 had almost undetectable histone deactylase activity *in vitro* but was active when transfected into cells promoted us to consider that SIRT6 perhaps required other partners for activity. To this end we performed a comprehensive yeast two-hybrid study using wild type full length SIRT6 as bait against a human monocyte/leukocyte library. This experiment identified three SIRT6 binding proteins that could reproducibly be shown to interact and moreover bound equally well to the catalytically dead H133W mutant. Thymine DNA glycosylase (TDG) is a mismatch specific DNA glycosylase that mediates the excision of mispaired thymines (G:T) and uracils (G:U) following spontaneous deamination of methylated cytosines to generate thymine. TDG excises the mismatched T or U to generate an abasic site that is subsequently repaired by base excision repair (BER) enzymes. SIRT6 has been linked to DNA repair and early studies suggested it played a role in BER. More recent studies have implicated SIRT6 in the non-homologous end joining (NHEJ) pathway of double strand break repair [11] and additionally it has been shown that SIRT6 mediates DSB repair through both non-homologous end joining and homologous recombination [7]. It was shown that these DSB repair functions of SIRT6 required both the histone deacetylase and ADP-ribosyltransferase activities and depended on the physical interaction and ADP-ribosylation of PARP1. Since PARP1 is implicated in both DSB repair and BER our identification of TDG as a binding partner of SIRT6 is further evidence that it interacts with the machinery of BER and strengthens its involvement in this process. TDG is also known to regulate gene expression through its interaction with the transcriptional co-activators CREB binding protein and CBP/p300 [29] and SRC-1 [30], the estrogen [31] and retinoic acid nuclear receptors [32] and it physically interacts with p53 and functions as a co-activator [33]. The DNA binding and function of TDG is regulated by phosphorylation and acetylation and the control of TDG may be critically important to the maintenance of DNA repair, CpG dinucleotides and epigenetic regulation [34]. Consequently its interaction with SIRT6 fits well with the observed biology of SIRT6 deficient cells. Demonstration of SIRT6-TDG interaction by co-immunoprecipitation was challenging and not always consistent. It is possible that this interaction requires a particular context of DNA damage to be revealed and functional experiments to confirm this interaction are necessary.

The second target identified as a SIRT6 binder in the yeast-2-hybrid screen was protein inhibitor of activated STAT1 (PIAS1). PIAS1 is a SUMO E3 ligase and is well known as a transcriptional regulator that can suppress immune responses by blocking the binding of STAT1 and NFκB to the promoters of target genes. PIAS1 binds to NFκB target genes in response to inflammatory stimuli such as TNFα following phosphorylation on Ser90 by IKKα [35] and blocks binding of the transcription factor to DNA. PIAS1 and PIASy are important negative regulators of NFκB and STAT1 and they can affect the magnitude and selectivity of the gene activation response [36]. In addition to its inhibitory effect, sumolyation by PIAS1 increases activity of the Gli transcription factor family [37] which was of interest given the enrichment of Gli-responsive genes induced by the H133W mutant seen in previous studies [17]. PIAS1 is also involved in epigenetic repression as *Pias1* deletion results in reduced H3K9 methylation and enhanced promoter accessibility [38]; interestingly this is the same histone residue that is deacetylated by SIRT6. Of interest is that once again a SIRT6 binding protein is also a known p53 binding protein [39]. However, perhaps the most interesting link is that SUMO 1,2 and 3 accumulate at sites of DSB and that this requires PIAS1 and 4. Furthermore PIAS1 and 4 accumulate at sites of DSB and promote DSB repair and through recruitment of RNF4 [40]–[42]. Given that SIRT6 is also recruited to chromatin following DNA damage and has been implicated in DSB [11] the direct interaction of SIRT6 and PIAS1 adds a new insight to the involvement of SIRT6 in the DSB repair mechanism.

The third SIRT6 interacting protein identified by the yeast two hybrid screen was a novel alternatively spliced form of TSPYL2 (gi:47076936) that has a predicted ORF of 287 aa, the first 269 of which are identical to the first 269 aa of the major splice form of TSPYL2. TSPLY2, also known as Cell Division Autoantigen 1 (CDA1) is a nuclear protein that forms part of the CASK/TRB1 transcriptional complex and is thought to play a role in nucleosome assembly and is involved in cell cycle regulation and chromatin remodeling [43]. TSPYL2 can arrest the cell cycle by regulating p53 protein levels through inactivation of MDM2 and transcriptional regulation of p21^Waf1/Cip1^ [44] and TSPYL2 knock out mice have been shown to have a defective G1 arrest upon DNA damage [45]. A further link to p53 is that TSPYL2 directly binds p53 in yeast-2-hybrid assays (Hybrigenics personal communication). The alternative splice transcript we have identified as a SIRT6 binder contains the N-terminal Pro-rich domain and basic domain and part of the NAP-domain and although the function of this alternative splice variant is unknown, the full length TSPYL2 transcript is clearly implicated in similar biological processes to SIRT6. Although we were unable to confirm TDG and TSPYL2 interactions with SIRT6 by co-immunoprecipititation in this study, they are high confidence yeast two hybrid hits as they are not frequently found false positives in a very large number of screens performed against these libraries. Our proteomics approach to identifying SIRT6 binding proteins identified two more novel partners, namely MYBBP1A and SMARCA5. MYBBP1A interacts directly with RelA/p65 and was shown to repress NFκB dependent reporter gene activity but did not repress its nuclear translocation nor DNA binding activity [18]. Once again a SIRT6 binding partner is linked to p53 biology as MYBBP1A is involved in p53 acetylation and accumulation following nucleolar disruption [46].

SMARCA5 (hSNF2H) was cloned as a human homologue of Drosophila ISWI and has 86% sequence homology to hSNF2L [47]. SMARCA5 is a helicase that possesses intrinsic ATP-dependent nucleosome-remodeling activity as part of the SWI/SNF complex. There are many described chromatin-remodelling complexes that perform a variety of functions in nuclear processes [48]. SMARCA5 is a component of the B-WICH complex, a chromatin remodelling complex that mobilizes nucleosomes and reconfigures irregular chromatin to a regular nucleosomal array structure. The B-WICH complex regulates the transcription of various genes, has a role in RNA polymerase I and RNA polymerase III transcription, mediates the histone H2AX phosphorylation at ‘Tyr-142’, and is involved in the maintenance of chromatin structures during DNA replication processes. The B-WICH complex also contains the WSTF transcription factor (William’s syndrome transcription factor) and a number of other nuclear proteins including MYBBP1A [49] and the WSTF-SMARCA5 complex has been shown to play a role in the maintenance of chromatin structures during DNA replication [50]. The identification of two components of the B-WICH complex binding to SIRT6 suggests that it may be a functional component of this complex. The B-WICH complex is also involved in regulating rDNA transcription and siRNA silencing of WSTF leads to a reduced level of 45S pre-rRNA. WSTF knock down results in a reduced level of acetylated H3, in particular H3K9-Ac at the rRNA promoter and along the gene [51]. Therefore there is the intriguing possibility that SIRT6 functions as an H3K9 deacetylase as part of a larger complex. Recently, SIRT7, which is closely related to SIRT6, has also been shown to also bind SMARCA5 and MYBBP1A and be a component of the B-WICH complex [52]. SIRT7 unlike SIRT6 resides mainly in the nucleolus and is associated with the RNA Pol I machinery and is required for rDNA transcription. Although SIRT6 is mainly found in the nucleoplasm, recent studies have suggested that it is enriched in the nucleolus in the G_1_ phase of the cell cycle [10].

These studies have generated further information with regards the interactions of SIRT6 with cellular component and suggests potential roles for SIRT6 within a number of biological functions. Although they do not resolve the controversy over the role of SIRT6 in regulating NFκB, through its direct binding to RelA/p65 and its interaction with known NFκB regulators such as PIAS1 and MYBBP1A it increases our confidence that SIRT6 and NFκB have a functional relationship that requires greater understanding.

## Materials and Methods

### Cell Transfection

HEK293 cells (obtained from ATCC as CRL-1573) were routinely transfected with wild type SIRT6 cDNA or mutant forms of SIRT6 cloned into a pcDNA3 expression vector using OptiMEM medium (Invitrogen) and FuGene HD transfection reagent (Roche) according to the manufacturer’s instructions.

### Confocal Microscopy and Immunocytochemistry

Confocal microscopy was carried out as previously described [17].

### Western Blotting

Transfected cells were washed twice in PBS after removal of growth media, pelleted by centrifugation followed by lysis in ice cold lysis buffer containing 20 mM Tris-HCl pH7.4, 10 mM KCl, 10 mM MgCl_2_, 2 mM EDTA, 10% glycerol (v/v), 1% Triton X-100 (v/v), protease inhibitor cocktail (Roche, 1 tablet per 50 ml). Cells were gently sonicated using a probe sonicator (MSE Soniprep 20% output). NaCl was added to final concentration of 420 mM and cells lysed for 1 hour on ice, followed by additional gentle sonication. Cell lysates were clarified by centrifugation at 13000 rpm at 4°C for 30 minutes. Protein concentration was determined using DC Protein kit (BioRad). 50 µg protein extract of each sample was analysed by SDS-PAGE (NuPAGE, Invitrogen) and transferred to PVDF membrane using semi-dry blotting apparatus. Proteins were detected using a variety of antibodies including anti-SIRT6 (Sigma S2197 or Bethyl Lab. A302-452A) or anti-Flag-M2 HRP (Sigma A8592) antibodies and ECL reagent (Pierce). The anti-MYBBP1A antibody was obtained from Bethyl Lab. (A301-328A), the anti-RelA/p65 from Thermo/Fisher (RB-1638) and the anti-PIAS antibody was obtained from Abgent (AP1242A). The anti-SMARCA5 antibody (HPA008751) was obtained from Sigma.

### Immunoprecipitation

Total cell lysate (1.70×10^7^ cells/ml) was incubated with either anti-Flag antibody covalently attached to Agarose (Sigma, A2220) or with anti-RelA/p65 antibody (Bethyl lab., A301-823A) bound to Protein G covalently attached to Agarose (GE healthcare,17-0618-01) for 1 hour at room temperature. After washing the resin with Lysis Buffer, excessive volumes of Tris buffered saline (TBS) and 20 mM Tris, pH7.4 buffer, containing 0.5 M NaCl, the proteins bound to the resin were eluted under non-denaturing condition with either Flag peptide or with detergent using reduced SDS sample buffer and analysed by SDS-PAGE, LC-MS/MS and Western blotting. Cross-linked beaded Agarose 6B or Protein G Sepharose 4 Fast Flow were used as a control resins. For MYBBP1A, RelA/p65, PIAS1 and SIRT6 immunoprecipitates SIRT6 total cell lysate was incubated with following antibodies bound to protein G Agarose overnight at 4°C: polyclonal rabbit anti MYBBP1A (Bethyl Lab A301-328A, lot 301-328A-1), rabbit anti RelA/p65 (Bethyl Lab A301-824A), anti PIAS1 (Abcam ab32219), anti SIRT6 (Bethyl LabA302-451A). After washing the resin with lysis buffer, TBS and then with 20 mM Tris, pH7.4 buffer, containing 0.5 M NaCl, proteins were eluted with reduced SDS sample buffer. As a “no antibody” control crosslinked Agarose 6B was used to adsorb the cell extract. Immunoprecipitated samples were run on 4–12% NuPAGE gels and transferred to Nitrocellulose membrane using an iBlot system. The membrane was blocked in 5% milk-PBS overnight at 4°C. SIRT6 was probed on the Western blot either with anti Flag antibody (SigmaA8592) or with anti SIRT6 antibody (Sigma S4322). For detection of the anti SIRT6 antibody following incubation with primary antibody the membrane was washed with Tween-TBS and incubated with fluorescent donkey anti rabbit IRD680LT (LI-COR) secondary antibody in Tween-PBS for 1 hr at room temperature protected from light then visualised using Odyssey Infrared Imaging System (LI-COR).

### Proteomics. Digestion of Gel Bands

Protein bands were excised from the polyacylamide gels, reduced with DTT, the cysteine residues carboxyamidated and digested *in situ* with trypsin according to a modification of previously published methods [53], [54].

The gel was rinsed with water and bands of interest were excised with a clean scalpel. Each band was then chopped into cubes (approximately 1 mm^3^) and the pieces transferred into a 0.5 ml microcentrifuge tube. The gel pieces were then washed with 150 µl water for 5 minutes on an orbital shaker, the tube spun and the liquid removed with a fine-bore long gel-loading pipette tip. Acetonitrile (50 µl) was then added for 10–15 minutes to allow the gel pieces to shrink and the tube again spun in order to remove the liquid. The sample was dried for 15 minutes in a vacuum centrifuge. The gel pieces were swollen in 50 µl of 1.5 mg/ml DTT, 0.1 M ammonium bicarbonate for 30 minutes at 56°C to reduce the protein. Following centrifugation, residual liquid was removed and acetonitrile (50 µl) added to shrink the gel pieces and the tube again spun in order to remove the liquid. The sample was again dried for 15 minutes in a vacuum centrifuge. The gel pieces were then swollen in 50 µl 10 mg/ml iodocetamide, 0.1 M ammonium bicarbonate for 20 minutes at room temperature in the dark, to derivatise the cysteine residues in the protein. Residual liquid was removed and the gel pieces washed with 200 µl 0.1 M ammonium bicarbonate for 15 minutes on an orbital shaker. Following centrifugation, residual liquid was removed and acetonitrile (50 µl) added to shrink the gel pieces for 10 minutes and the tube again spun in order to remove the liquid. The sample was finally dried for 30 minutes in a vacuum centrifuge. The dry gel pieces were then swollen in 15–20 µl of digestion buffer (50 mM ammonium bicarbonate, 5 mM calcium chloride) containing 13 ng/ml of trypsin for 45 minutes on ice. The tube was then centrifuged and any residual unabsorbed liquid carefully removed to reduce excess amounts of trypsin. Finally, 10 µl digestion buffer was added and the sample digested at 37°C overnight. After the overnight incubation, the tube was spun to dislodge water droplets condensed on the tube lid and the sample left for 10 minutes for the gel pieces to reabsorb the liquid.

### Preparation of Sample for MALDI-TOF MS Analysis

A 1 µl aliquot of the liquid surrounding the gel pieces was mixed with 1 µl matrix solution (60% acetonitrile (v/v), 0.5% trifluoroacetic acid (v/v), 6 mg/ml α-cyano-4-hydroxycinnamic acid) in the bottom of a microfuge tube. Immediately, 1 µl of the mixture was spotted onto a 384-well stainless steel MALDI-TOF mass spectrometer target and allowed to dry, leaving a crystallized mixture of sample and matrix on the target for analysis. Peptide calibration standard solution (Bruker Daltonics, Bremen, Germany) was spotted in a similar manner adjacent to the samples.

### MALDI-TOF Mass Spectrometry

Matrix-assisted laser-desorption/ionization time-of-flight mass spectra were collected on an Ultraflex III mass spectrometer (Bruker Daltonics, Bremen, Germany) in reflectron mode. Individual peptide ions were selected for fragmentation analysis by LIFT-MS/MS sequencing [55]. Spectra were interpreted using FlexAnalysis and Biotools software (Bruker Daltonics) and the data searched against protein sequence databases using the program Mascot [56].

### LC-ESI Mass Spectrometry

Digest (5 µl) was diluted with 5 µl 0.1% formic acid (v/v) and 1 µl injected onto an Agilent HPLC coupled to a Bruker HCT+ Ultra ion-trap mass spectrometer. The sample was separated on a reversed-phase column (Waters 3.5 µm Xbridge BEH130 C18, 300 µm×150 mm) by gradient elution using a 1 hour gradient (60 min_capillary_lcms.m) from 2% acetonitrile (v/v), 0.1% formic acid (v/v) to 60% acetonitrile (v/v), 0.1% formic acid (v/v). Automatic ms and ms/ms peak selection was used and the data collected between 2–45 minutes were used to generate compound lists for database searching. Several blank injections of 0.1% formic acid (v/v) were performed at the start of a sample batch to wash any impurities/peptides that could have accumulated on the column in the period it had been left unused. A blank containing 0.1% formic acid (v/v), 50% acetonitrile (v/v) was injected between each run to elute any residual bound material off the column from the previous sample. The detector and mass calibration was carried out monthly by injecting 0.5 ml tune mix diluted 1∶50 with acetonitrile by means of a syringe pump.

### Yeast Two-Hybrid Analysis

Yeast two-hybrid screening was performed by Hybrigenics, S.A., Paris, France (http://www.hybrigenics-services.com). The coding sequence for full-length SIRT6 (GenBank accession number gi:7706709) was PCR-amplified and cloned into pB27 as a C-terminal fusion to LexA (N-LexA-SIRT6-C) and into pB35 as a C-terminal fusion to Gal4 DNA-binding domain (N-Gal4-SIRT6-C). The constructs were checked by sequencing the entire insert and used as a bait to screen a random-primed human leukocyte and activated mononuclear cell cDNA library constructed into pP6. pB27 and pP6 derive from the original pBTM116 [57] and pGADGH [58] plasmids, respectively. pB35 was constructed by inserting the Gal4 DNA-binding domain from pAS2ΔΔ [59] into the pFL39 backbone [60] under the control of MET25 promoter [61]. For the LexA bait construct, 91 million clones (9-fold the complexity of the library) were screened using a mating approach with Y187 (matα) and L40ΔGal4 (mata) yeast strains as previously described (53). A total of 32 His+ colonies were selected on a medium lacking tryptophan, leucine and histidine. For the Gal4 construct, 85 million clones (8-fold the complexity of the library) were screened using the same mating approach with Y187 (matα) and CG1945 (mata) yeast strains. A total of 22 His+ colonies were selected on a medium lacking tryptophan, leucine, histidine and methionine. The prey fragments of the positive clones were amplified by PCR and sequenced at their 5′ and 3′ junctions. The resulting sequences were used to identify the corresponding interacting proteins in the GenBank database (NCBI) using a fully automated procedure. A confidence score (PBS, for Predicted Biological Score) was attributed to each interaction as previously described [62].

## Supporting Information

Figure S1 **Alignment of peptides identified by MS sequencing to the cDNA sequences of MYBBP1A and SMARCA5.** Identified peptides are shown in red bold type.
(DOCX)
