# Supplementary material for: Identification of Novel Interacting Partners of Sirtuin6
Source: PLoS One. 2012 Dec 11;7(12):e51555. doi: 10.1371/journal.pone.0051555 (PMC3519869; doi:10.1371/journal.pone.0051555)
Supplement: Figure S1 — Alignment of peptides identified by MS sequencing to the cDNA sequences of MYBBP1A and SMARCA5. Identified peptides are shown in red bold type. (DOCX) [file pone.0051555.s001.docx]

**Supplementary Figure 1**. Alignment of peptides identified by MS sequencing to the cDNA sequences of MYBBP1A and SMARCA5. Identified peptides are shown in red bold type.

Q9BQG0|MBB1A_HUMAN Myb-binding protein 1A OS=Homo sapiens GN=MYBBP1A PE=1 SV=2

MESRDPAQPMSPGEATQSGARPADRYGLLKHSREFLDFFWDIAKPEQETRLAATEKLLEY

LRGRPKGSEMKYALKR**LITGLGVGR**ETARPCYSLALAQLLQSFEDLPLCSILQQIQEKYD

LHQVKKAMLRPALFANLFGVLALFQSGRLVKDQEALMKSVKLLQALAQYQNHLQEQPRKA

LVDILSEVSKATLQEILPEVLKADLNIILSSPEQLELFLLAQQKVPSKLKKLVGSVNLFS

DENVPRLVNVLKMAASSVKKDRKLPAIALDLLRLALKEDKFPRFWKEVVEQGLLKMQFWP

ASYLCFRLLGAALPLLTKEQLHLVMQGDVIRHYGEHVCTAKLPKQFKFAPEMDDYVGTFL

EGCQDDPERQLAVLVAFSSVTNQGLPVTPTFWRVVRFLSPPALQGYVAWLRAMFLQPDLD

SLVDFSTNNQKKAQDSSLHMPERAVFRLRKWIIFRLVSIVDSLHLEMEEALTEQVARFCL

FHSFFVTKKPTSQIPETKHPFSFPLENQAREAVSSAFFSLLQTLSTQFKQAPGQTQGGQP

WTYHLVQFADLLLNHSHNVTTVTPFTAQQRQAWDRMLQTLKELEAHSAEARAAAFQHLLL

LVGIHLLKSPAESCDLLGDIQTCIRKSLGEKPRRSRTKTIDPQEPPWVEVLVEILLALLA

QPSHLMRQVARSVFGHICSHLTPRALQLILDVLNPETSEDENDR**VVVTDDSDER**RLKGAE

DKSEEGEDNRSSESEEESEGEESEEEERDGDVDQGFREQLMTVLQAGKALGGEDSENEEE

LGDEAMMALDQSLASLFAEQKLRIQARRDEKNKLQKEKALRRDFQIRVLDLVEVLVTKQP

ENALVLELLEPLLSIIRRSLRSSSSKQEQDLLHKTARIFTHHLCRARRYCHDLGERAGAL

HAQVERLVQQAGRQPDSPTALYHFNASLYLLRVLKGNTAEGCVHETQEKQKAGTDPSHMP

TGPQAASCLDLNLVTRVYSTALSSFLTKRNSPLTVPMFLSLFSRHPVLCQSLLPILVQHI

TGPVRPRHQACLLLQKTLSMREVRSCFEDPEWKQLMGQVLAKVTENLRVLGEAQTKAQHQ

QALSSLELLNVLFRTCKHEKLTLDLTVLLGVLQGQQQSLQQGAHSTGSSRLHDLYWQAMK

TLGVQRPKLEKKDAK**EIPSATQSPISK**KRKKKGFLPETKKRKKR**KSEDGTPAEDGTPAAT**

**GGSQPPSMGR**KKRNRTKAKVPAQANGTPTTKSPAPGAPTRSPSTPAKSPKLQKKNQKPSQ

VNGAPGSPTEPAGQKQHQKALPKKGVLGK**SPLSALAR**KKARLSLVIR**SPSLLQSGAK**KKA

QVRKAGKP

O60264|SMCA5_HUMAN SWI/SNF-related matrix-associated actin-dependent regulator of chromatin subfamily A member 5 OS=Homo sapiens GN=SMARCA5 PE=1 SV=1

MSSAAEPPPPPPPESAPSKPAASIASGGSNSSNKGGPEGVAAQAVASAASAGPADAEMEE

IFDDASPGKQK**EIQEPDPTYEEK**MQTDRANRFEYLLKQTELFAHFIQPAAQKTPTSPLKM

KPGRPRIKKDEKQNLLSVGDYRHR**RTEQEEDEELLTESSK**ATNVCTRFEDSPSYVKWGKL

RDYQVRGLNWLISLYENGINGILADEMGLGKTLQTISLLGYMKHYRNIPGPHMVLVPKST

LHNWMSEFKRWVPTLRSVCLIGDKEQRAAFVRDVLLPGEWDVCVTSYEMLIKEKSVFKKF

NWRYLVIDEAHRIKNEKSKLSEIVREFKTTNRLLLTGTPLQNNLHELWSLLNFLLPDVFN

SADDFDSWFDTNNCLGDQKLVERLHMVLRPFLLRRIKADVEKSLPPKKEVKIYVGLSKMQ

REWYTRILMKDIDILNSAGKMDKMRLLNILMQLRKCCNHPYLFDGAEPGPPYTTDMHLVT

NSGKMVVLDKLLPKLKEQGSRVLIFSQMTRVLDILEDYCMWRNYEYCRLDGQTPHDERQD

SINAYNEPNSTKFVFMLSTRAGGLGINLATADVVILYDSDWNPQVDLQAMDRAHRIGQTK

TVRVFRFITDNTVEERIVERAEMKLRLDSIVIQQGR**LVDQNLNK**IGKDEMLQMIRHGATH

VFASK**ESEITDEDIDGILER**GAKKTAEMNEKLSKMGESSLRNFTMDTESSVYNFEGEDYR

EKQKIAFTEWIEPPKRERKANYAVDAYFREALRVSEPKAPKAPRPPKQPNVQDFQFFPPR

LFELLEKEILFYRKTIGYKVPRNPELPNAAQAQKEEQLKIDEAESLNDEELEEKEKLLTQ

GFTNWNKRDFNQFIKANEKWGRDDIENIAREVEGKTPEEVIEYSAVFWERCNELQDIEKI

MAQIERGEARIQRRISIKKALDTKIGRYKAPFHQLRISYGTNKGKNYTEEEDRFLICMLH

KLGFDKENVYDELRQCIRNSPQFRFDWFLKSRTAMELQRRCNTLITLIERENMELEEKEK

AEKKKRGPKPSTQKRKMDGAPDGRGRKKKLKL
